# Altered Intracortical Inhibition in Chronic Traumatic Diffuse Axonal Injury

**DOI:** 10.3389/fneur.2018.00189

**Published:** 2018-03-28

**Authors:** Cintya Yukie Hayashi, Iuri Santana Neville, Priscila Aparecida Rodrigues, Ricardo Galhardoni, André Russowsky Brunoni, Ana Luiza Zaninotto, Vinicius Monteiro de Paula Guirado, Ana Sofia Cueva, Daniel Ciampi de Andrade, Manoel Jacobsen Teixeira, Wellingson Silva Paiva

**Affiliations:** ^1^Department of Neurology, School of Medicine, University of São Paulo (USP), São Paulo, Brazil; ^2^School of Medicine, Universidade da Cidade de São Paulo (UNICID), São Paulo, Brazil; ^3^Service of Interdisciplinary Neuromodulation, Institute of Psychiatry, Hospital das Clínicas HCFMUSP, University of São Paulo (USP), São Paulo, Brazil; ^4^Division of Psychology, Hospital das Clínicas HCFMUSP, University of São Paulo (USP), São Paulo, Brazil

**Keywords:** brain injuries, craniocerebral trauma, diffuse axonal injury, neurophysiology, transcranial magnetic stimulation

## Abstract

**Background:**

Overactivation of NMDA-mediated excitatory processes and excess of GABA-mediated inhibition are attributed to the acute and subacute phases, respectively, after a traumatic brain injury (TBI). However, there are few studies regarding the circuitry during the chronic phase of brain injury.

**Objective:**

To evaluate the cortical excitability (CE) during the chronic phase of TBI in victims diagnosed with diffuse axonal injury (DAI).

**Methods:**

The 22 adult subjects were evaluated after a minimum of 1 year from the onset of moderate or severe TBI. Each of the subjects first had a comprehensive neuropsychological assessment to evaluate executive functions—attention, memory, verbal fluency, and information processing speed. Then, CE assessment was performed with a circular coil applying single-pulse and paired-pulse transcranial magnetic stimulation over the cortical representation of the abductor pollicis brevis muscle on M1 of both hemispheres. The CE parameters measured were resting motor threshold (RMT), motor-evoked potentials (MEPs), short-interval intracortical inhibition (SIICI), and intracortical facilitation (ICF). All data were compared with that of a control group that consisted of the healthy age-matched individuals.

**Results:**

No significant differences between the left and right hemispheres were detected in the DAI subjects. Therefore, parameters were analyzed as pooled data. Values of RMT, MEPs, and ICF from DAI patients were within normal limits. However, SIICI values were higher in the DAI group—DAI SIICI = 1.28 (1.01; 1.87) versus the control value = 0.56 (0.33; 0.69)—suggesting that they had a disarranged inhibitory system (*p* < 0.001). By contrast, the neuropsychological findings had weak correlation with the CE data.

**Conclusion:**

As inhibition processes involve GABA-mediated circuitry, it is likely that the DAI pathophysiology itself (disruption of axons) may deplete GABA and contribute to ongoing disinhibition of these neural circuits of the cerebrum during the chronic phase of DAI.

## Introduction

Traumatic brain injury (TBI) is one of the major health consequences of trauma from motor vehicle accidents having its highest incidence among young male adults (1, 2). Considering that this age group is at its prime economic productivity, should they suffer a TBI which can cause long-term motor and cognitive disabilities, the negative impact of TBI stretches beyond just the individual victim (3, 4).

Traumatic brain injury is classified by clinical severity (mild, moderate, or severe), pathoanatomic type (focal or diffuse), and mechanism of injury (blunt or penetrating) (1). Focal lesions tend to have simpler management compared to diffuse injuries, whereas widespread damage has a complex mechanism that contributes to morbidity and limits clinical study and management.

Diffuse axonal injury (DAI) is a clinical condition often related to closed head traumas. It is the predominant finding in approximately half of TBI patients, and it has been found in all levels of TBI severity. Clinically, trauma victims with prolonged unconsciousness (6 h or more) unaccompanied by ischemic damage or mass lesions are usually diagnosed with DAI. Still, most of the features of DAI are microscopic and cannot be identified on conventional methods of neuroimaging, such as computed tomography (CT) scans or conventional magnetic resonance imaging (MRI) (1, 5, 6).

The diagnosis of DAI can only be confirmed by postmortem histopathological analysis and, for this reason, the development of new and more refined techniques—for instance, diffusion-weighted imaging and diffusion tensor imaging in MRI—enables further studies of DAI *in vivo*. In particular, transcranial magnetic stimulation (TMS) seems to be an interesting tool for neurophysiological testing as it allows a noninvasive real-time study of the brain, providing indirect information about intracortical interneuronal circuits through cortical excitability (CE) assessments (6–10).

Some studies using TMS show changes in CE after strokes, in psychiatric disorders, in pain syndromes, and even during acute phases of TBI (11–14). Mechanisms of intracortical facilitation (ICF) and intracortical inhibition are related to glutamatergic and GABAergic pathways, respectively, and the imbalance of these neurotransmitters is somehow involved in maladaptive plasticity (15, 16). Knowledge about how these imbalances lead to the pathophysiology that develops after brain injury would enable the development of new therapeutic strategies and rehabilitation options.

Unfortunately, there are few studies of CE after TBI, and most of those studies were carried out in patients with TBI of mild severity or only during the acute phase (14–16). Thus, in the present study, we sought to evaluate CE in patients during the chronic phase of TBI, diagnosed with DAI, using the diagnostic mode of TMS. In addition, we tried to correlate the neuropsychological profile of these patients with the CE data and clinical characteristics.

## Materials and Methods

### Setting and Subjects

A convenience sample of 73 adults, 18–60 years old, of both genders, who had been clinically diagnosed with DAI during an acute hospitalization following trauma were initially evaluated at the neurotrauma outpatient center of a tertiary referral hospital in Sao Paulo, Brazil.

After this initial screening, 51 patients were excluded. Exclusion criteria included (1) associated focal lesions or the presence of any abnormality other than DAI (e.g., epidural/subdural hematoma); (2) having suffered more than one TBI; (3) major psychiatric disorders (e.g., major depression, bipolar disorder, any disorder requiring admission to a psychiatric ward); (4) history of surgical procedures to the brain/skull; (5) the presence of metallic devices/pieces in the brain/skull (clips, plates, electrodes, etc.); (6) pregnancy; (7) epilepsy/seizures; (8) severe language impairment (writing/reading/speaking); or (9) illicit drug and/or alcohol abuse.

To achieve the most homogeneous sample possible, DAI diagnosis was established for this study as (1) a clinical condition of prolonged unconsciousness (6 h or more) following TBI; (2) a head CT scan image taken during acute hospitalization, demonstrating a wide spectrum of findings such as a relatively normal examination, small hemorrhagic (hyperdense) or non-hemorrhagic (hypodense) lesions no more than 25 cm^3^ in size (typically located at the gray–white matter junction, in the corpus callosum, and in more severe cases in the brainstem); small intraventricular hemorrhage, subarachnoid hemorrhage, and signs of brain swelling, such as compressed or even absent basal cisterns; (3) MRI obtained during the chronic phase (i.e., taken at least 6 months after TBI), demonstrating small regions of susceptibility artifact at the gray–white matter junction, in the corpus callosum, or the brain stem. Some lesions might be entirely non-hemorrhagic (even using susceptibility-weighted imaging sequences at high-field strengths). These would, however, be visible as regions of high fluid-attenuated inversion recovery signals on an MRI of the cranium.

Twenty-two subjects selected to participate in this exploratory study were assessed after a 1-year interval, at least, from the moment of trauma. The recruitment period was from May 2014 to 2015. For general comparisons, we used the normative data of CE as reference (17), and for supplementary analysis, we selected a control group consisting of 22 healthy subjects, with no history of brain injury or trauma, from a normative CE database (17), matching the DAI subjects for age and gender.

The protocol was approved by the Ethics Committee for Research of the respective institutions (Protocol #707.642), in compliance with the Declaration of Helsinki, and written informed consent was obtained from all the subjects participating in the study.

### Neuropsychological Assessment

All of the selected patients underwent a broad neuropsychological assessment to evaluate attention, memory, information processing speed, dexterity, and executive functions (inhibitory control, verbal fluency, and working memory). All the neuropsychological tests were conducted at one session in a quiet room, with only the subject and the examiner, at 2–7 days before the CE assessment and included the following:

– STAI (State-Trait Anxiety Inventory) (18, 19),
– BDI (Beck Depression Inventory) (18, 20),
– HVLT (Hopkins Verbal Learning Test)—immediate recall/delayed recall/recognition (21),
– BVMT (Brief Visual Memory Test)—immediate recall/delayed recall/recognition (21),
– TMT A and B (Trail-Making Test parts A and B) focused visual attention and task-switching attention (22),
– Stroop test, Victoria version—selective attention and inhibition (22),
– Digit Span Test—working memory (23),
– COWAT (Controlled Oral Word Association Test)—phonologic and semantic verbal fluency (24),
– Symbol digit test—information processing speed (24),
– Five-point test—visual fluency (24),
– Grooved pegboard test—dexterity (25).

### CE Assessment: TMS

The CE assessment was performed using MagPro X100 (MagVenture Tonika Elektronik, Farum, Denmark) with a C-100 circular coil connected to an electromyography amplifier of a one-channel, three-surface electrode output.

The stimulation target—*hotspot*—was determined using 70% intensity to identify the point of highest response of the hand muscles, which would correspond to the cortical representation of the abductor pollicis brevis muscle on M1 of both hemispheres. Each subject sat comfortably on a reclining armchair and wore a polyester swim cap on which the *hotspot* was marked.

The parameters measured were resting motor threshold (RMT), motor-evoked potentials (MEPs), short-interval intracortical inhibition (SIICI), and ICF. Peak-to-peak MEP amplitudes were considered in microvolts (μV). RMT was established as the lowest intensity at which MEP of at least 50-µV amplitude could be elicited in 5 of 10 consecutive stimuli (13, 17, 26–30).

We used single-pulse TMS for RMT and MEP measurements. The average value of four MEP curves taken at 120% of RMT was used for analysis. The same procedure was adopted for MEP curves at 140% of RMT. Paired-pulse TMS (pp-TMS) was used for SIICI and ICF measurements, with the conditioning stimulus set at 80% of RMT and the test stimulus at 120% of RMT (13). For SIICI analysis, response curves were taken using pp-TMS with 2 and 4 millisecond (ms) intervals between pulses [interstimulus intervals (ISI)], denominated ICI 2 ms and ICI 4 ms. As for ICF, ISI were 10 and 15 ms, denominated ICI 10 ms and ICI 15 ms. The average value of four MEP curves at each interval was used for analysis. CE measurements were performed using the same technique as in previous studies to facilitate comparison (17, 26–30).

### Statistical Analysis

All neuropsychological and CE data were analyzed using the SPSS version 22.0 Statistical package (SPSS, IBM Inc., Chicago, IL, USA) with two-tailed tests and a 5% level of significance. Shapiro–Wilk tests were used to verify continuous variables for normal distribution, and Wilcoxon tests were used to compare the right and left hemispheres in the DAI group.

For inferential analysis, all subjects from DAI group were matched by age and gender to healthy subjects from a normative database of CE (17), and a Mann–Whitney *U*-test was performed. The Spearman test was performed to analyze correlation between neuropsychological and CE data results.

## Results

### Demographic and Clinical Characteristics

Most participants had severe TBI. They were mostly young adult males (86%), as trauma in general is common in this group. For the outcome measure (functionality), participants were classified according to the Glasgow Outcome Scale-Extended (31). Most of them were independent, both inside and outside their homes but could not resume all their pre-injury social activities—*upper moderate disability*—mostly due to irritability, concentration problems, and memory failures (Table 1).

**Table 1:** Demographic and clinical characteristics of 22 subjects with DAI.

	Mean (±SD) or number (%)
**Demographic**	
Age, years	30.1 (±10.3)
Gender, male	19 (86.36)
Education, years	10.3 (±2.3)
**Clinical characteristics**	
Handedness, right	20 (90.9)
Time since TBI, months	18.7 (±2.5)
Glasgow Outcome Scale-Extended	
*5—Lower Moderate Disability*	2 (9.09)
*6—Upper Moderate Disability*	10 (45.45)
*7—Lower Good Recovery*	7 (31.82)
*8—Upper Good Recovery*	3 (13.64)
Glasgow Coma Scale, score <8 at admission	16 (72.73)
Mechanisms of injury	
*Automobile accident*	9 (40.91)
*Motorcycle accident*	8 (36.36)
*Running-over*	3 (13.64)
*Interpersonal aggression*	2 (9.09)
Medication	
*None*	19 (86.36)
*Benzodiazepine (prior use)*	2 (9.09)
*Antidepressant (prior use)*	1 (4.55)

Three subjects had used medications in the past that could interfere with the neurophysiological tests, but by the time they were included in the study, they were no longer taking any medications and were not outliers on CE data. Therefore, they were not excluded from analysis.

### CE Results

The CE data distribution was skewed, and there were no significant differences between hemispheres (*p* = 0.125). A pooled analysis was performed in which data from both hemispheres (left and right) were combined (22 subjects, 44 hemispheres). One subject had traumatic amputation of the right arm, and CE data of the left hemisphere were not collected. Another subject had a brachial plexus injury of the left arm, and CE data of right hemisphere were not collected. In summary, 42 hemispheres were analyzed for the DAI patients.

#### DAI Group Data Classified According to Normative Data

On a group analysis, CE values for the DAI group were classified according to normative data (Table 2). The confidence interval range of normal values obtained by Cueva and collaborators (17) was utilized to classify CE results for each parameter of each patient. Values above the highest confidence limit were classified as “high,” those within the confidence interval were classified as “normal,” and those under the lowest limit were classified as “low.”

**Table 2:** CE data comparison between DAI patients and healthy controls, analyzed according to normative data.

CE parameters	Median (95% CI)		*p*-Value	95% CI normative data (17)^a^	Classification of DAI patients*n*(%)		
	Control (*n* = 44)	DAI (*n* = 42)			High	Normal	Low
RMT	48.5 (44; 52)	47.5 (43; 51)	0.604	46.3–49.8	19 (45.24)	3 (7.14)	20 (47.62)
MEP-120%	310.5 (260.03; 436.52)	435.85 (253.38; 581.28)	0.388	423.3–689.6	11 (26.19)	11 (26.19)	20 (47.62)
MEP-140%	756.5 (592.31; 1,300)	1,000 (742.02; 1,385.64)	0.346	987.0–1,385.7	15 (35.71)	9 (21.43)	18 (42.86)
Ratio 140/120	2.02 (1.80; 3.16)	2.46 (1.79; 3.12)	0.853	2.4–3.3	11 (26.19)	11 (26.19)	20 (47.62)
ICI 2 ms	0.26 (0.20; 0.47)	1.28 (0.79; 1.70)	**<0.001**	0.2–0.4	**36 (85.71)**	5 (11.90)	1 (2.38)
ICI 4 ms	0.36 (0.28; 0.62)	1.17 (1.0; 1.84)	**<0.001**	0.4–0.6	**36 (85.71)**	5 (11.90)	1 (2.38)
ICF 10 ms	1.31 (0.91; 1.77)	1.20 (0.94; 1.57)	0.638	1.5–2.1	7 (16.67)	10 (23.81)	25 (59.52)
ICF 15 ms	1.15 (0.87; 1.37)	1.47 (1.18; 1.88)	0.131	1.4–2.1	12 (28.57)	11 (26.19)	19 (45.24)
SIICI	0.56 (0.33; 0.69)	1.28 (1.01; 1.87)	**<0.001**	0.4–0.6	**36 (85.71)**	4 (9.52)	2 (4.76)
ICF	1.13 (0.96; 1.48)	1.40 (1.10; 1.68)	0.432	1.5–2.0	10 (23.81)	10 (23.81)	22 (52.38)

*^a^Normative data 95% CI obtained by Cueva (17)*.

*RMT, Resting motor threshold; MEP-120%, Motor-evoked potential at 120% of RMT; MEP-140%, Motor-evoked potential at 140% of RMT; Ratio 140/120, Motor-evoked potential amplitude ratio for stimulus intensity at 140 and 120% of RMT; ICI 2 ms, Motor-evoked potential amplitude ratio for 2 ms ISI and 120% of RMT; ICI 4 ms, Motor-evoked potential amplitude ratio for 4 ms ISI and 120% of RMT; ICF 10 ms, Motor-evoked potential amplitude ratio for 10 ms ISI and 120% of RMT; ICF 15 ms, Motor-evoked potential ratio for 15 ms ISI and 120% of RMT; SIICI, Short-interval intracortical inhibition; ICF, intracortical facilitation; CI, confidence interval*.

For each CE parameter, there were patients with higher/lower values than normative data ones. For RMT values, there were 39 hemispheres (92.86%) out of common healthy values (normative data); for MEP-120% values, there were 31 hemispheres (73.81%); for MEP-140%, there were 33 hemispheres (78.57%); for Ratio 140/120, there were 31 hemispheres (73.81%); for ICI 2 ms, there were 37 hemispheres (88.09%); for ICI 4 ms, there were also 37 hemispheres (88.09%); for ICF 10 ms, there were 32 (76.19%); for ICF 15 ms, there were 31 (73.81%); for SIICI, there were 38 (90.47%), and for ICF, there were 32 (76.19%).

#### DAI Patients Compared to Healthy Controls

**Figure 1:**
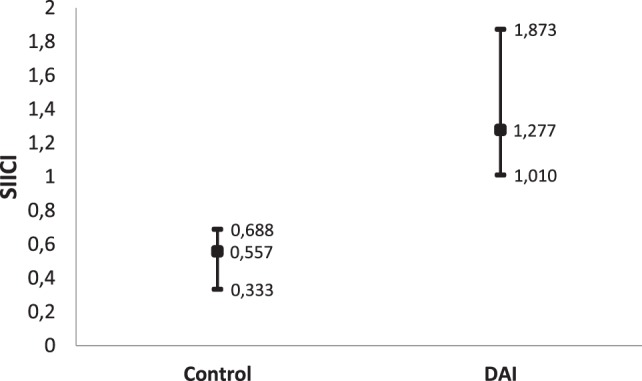
Short-interval intracortical inhibition (SIICI) median values and 95% CI of diffuse axonal injury (DAI) patients compared to healthy subjects.

When data from the DAI patients on an individual level were analyzed, the difference on SIICI and its components was indeed statistically significant compared to those of healthy subjects (Table 2). Normal SIICI values usually range from 0.0 to 1.0, and our results showed SIICI median values of 1.28 (1.01; 1.87) (Figure 1).

#### Neuropsychological Results

The majority of neuropsychological tests of patients with DAI showed mean raw scores below the average expected for healthy individuals in our country. Assessments of HVLT, BVMT (immediate and delayed recall), TMT, Stroop, COWAT, Symbol Digit, Five Points, and Grooved Pegboard indicated that DAI patients were cognitively impaired considering age and/or years of schooling. On the other hand, full Digit Span test and only the recognition part of BVMT showed results within the normative average. Table 3 presents mean scores of neuropsychological assessment. Each test has a standard score limit that defines normal and/or impaired function.

**Table 3:** Results of the neuropsychological tests and inventories.

Tests/inventory		*n*	Mean Raw score (SD)	Mean *Z*-score (SD)
STAI	–	21	54.14 (8.79)	–
BDI	–	21	14.19 (12.03)	–
HVLT	Immediate recall	22	17.68 (4.19)	−1.79 (1.06)
	Delayed recall	22	5.23 (2.79)	−1.42 (1.21)
	Recognition	22	9.86 (1.78)	0.75 (1.30)
BVMT	Immediate recall	21	16.38 (8.65)	−1.10 (1.17)
	Delayed recall	21	6.76 (4.23)	−1.13 (1.47)
	Recognition	21	5.24 (1.09)	−0.44 (1.18)
TMT	Part A	22	49.72 (27.12)^a^	−0.80 (1.39)
	Part B	20	135.55 (91.36)^a^	−1.43 (1.22)
Stroop	Card 1	22	23.90 (14.88)^a^	−1.80 (1.25)
	Card 2	22	24.86 (10.22)^a^	−1.35 (1.20)
	Card 3	22	34.09 (11.88)^a^	−1.17 (1.41)
Digit Span	Original order	22	5.00 (1.07)	−0.01 (0.77)
	Reversed order	22	3.45 (0.80)	−0.42 (0.50)
COWAT	Phonologic	22	24.14 (9.77)	−1.48 (0.84)
	Semantic	21	14.38 (4.65)	−1.39 (0.97)
Symbol Digit	–	22	38.45 (14.29)	−1.86 (1.00)
Five Point	–	22	19.64 (7.21)	−1.47 (0.87)
Grooved	Dominant hand	19	87.42 (22.80)^a^	−1.43 (1.27)
Pegboard	Non-dominant hand	21	108.10 (35.89)^a^	−1.70 (1.15)

*^a^Task execution score measured by time in seconds*.

*BDI, Beck Depression Inventory; BVMT, Brief Visual Memory Test; COWAT, Controlled Oral Word Association Test; HVLT, Hopkins Verbal Learning Test; STAI, State-Trait Anxiety Inventory; TMT, Trail-Making Test*.

**Figure 2:**
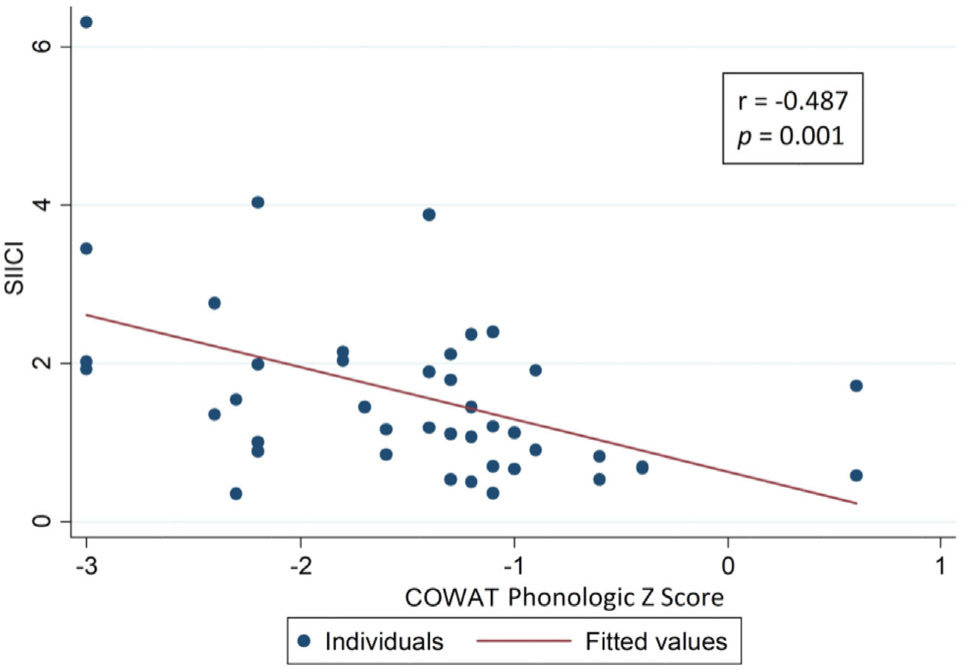
Correlation between short-interval intracortical inhibition (SIICI) values and Controlled Oral Word Association Test (COWAT) phonologic test results.

**Figure 3:**
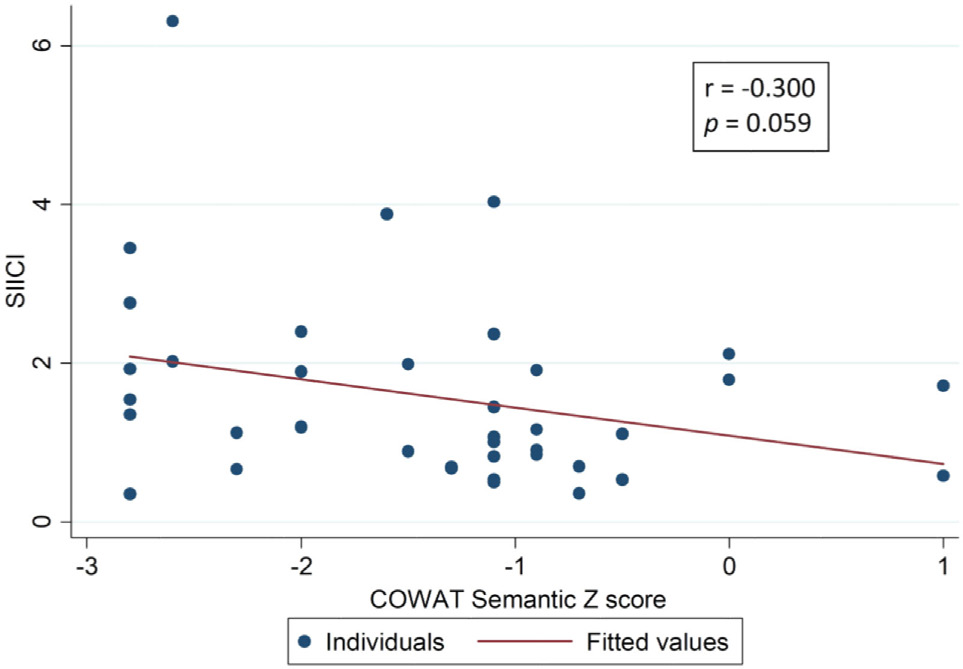
Correlation between short-interval intracortical inhibition (SIICI) values and Controlled Oral Word Association Test (COWAT) semantic test results.

**Figure 4:**
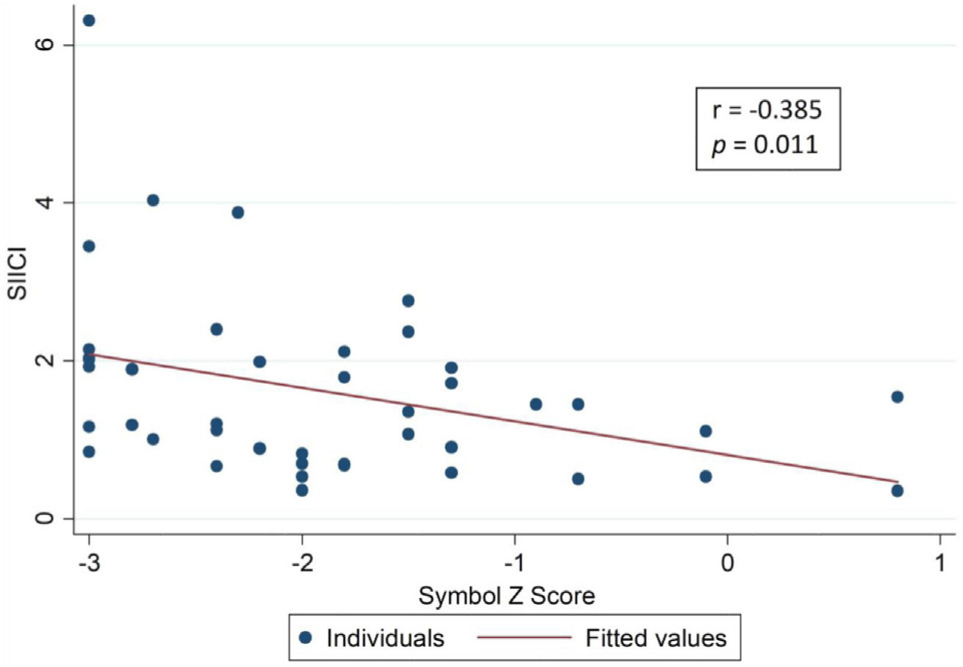
Correlation between short-interval intracortical inhibition (SIICI) values and Symbol Digit test results.

The hypothesis from the SIICI information and neuropsychological findings was that both of these could somehow be related *via* effects on inhibitory processes. For that reason, a correlation analysis was attempted using tests that assessed selective attention and inhibition (Table 4), but only weak correlations were found, though, and few of them were statistically significant (Figures 2–4).

**Table 4:** Correlation between SIICI values and inhibitory control main neuropsychological test results (Spearman’s correlation coefficient).

Neuropsychological tests (inhibitory control assessment)		SIICI			
		rho	*p*-Value
Stroop Card 3	Time	0.080	0.611
	*Z*-score	−0.019	0.904
COWAT phonologic	Raw score	−0.407	**0.007**
	*Z*-score	−0.487	**0.001**
COWAT semantic	Raw score	−0.314	**0.047**
	*Z*-score	−0.300	**0.059**
Symbol digit	Raw score	−0.274	0.078
	*Z*-score	−0.385	**0.011**
Five point	Raw score	−0.129	0.414
	*Z*-score	−0.129	0.414
Grooved Pegboard dominant hand	Time	0.212	0.207
	*Z*-score	−0.151	0.370
Grooved Pegboard Non-dominant hand	Time	0.031	0.845
	*Z*-score	−0.121	0.449

*SIICI, Short-interval intracortical inhibition; COWAT, Controlled Oral Word Association Test*.

## Discussion

From a pathophysiologic perspective on DAI, damage to axons occurs at the moment of trauma (primary axotomy) when abrupt acceleration–deceleration of the cranium results in shear forces and tensile strains on the white matter, generating small loci of hemorrhage. The resulting axonal degeneration can last for many hours after the trauma episode (caused by secondary axotomy and biochemical cascades) (1, 5, 6).

To minimize the influence of inflammation on these neurophysiological changes, and focus on the direct neuronal damage, we selected subjects during the chronic phase of DAI (at least 1 year after TBI). Although there are many mechanisms involved in the development and evolution of DAI, such as diffuse vascular injury and blood–brain barrier disruption that could also interfere with the function of the neural circuitry, the paucity of studies on neurophysiological changes after moderate and severe DAI prevents comparison.

From the CE assessment perspective, pp-TMS using short ISI applied to the motor cortex can indirectly evaluate inhibitory processes mediated by GABAergic circuits (26). The altered SIICI results we found drove us to consider a possible association of it with dexterity alterations (Grooved Pegboard Test scores) as specific motor task controlling involves intracortical inhibition process by activating a few selective cells and barring other motor neurons (32). The statistical analysis showed that it was not significant even though patients with TBI often present associated motor and cognitive injuries due to the various mechanisms of trauma (4, 33, 34).

Only few neuropsychological tests showed results within the average expected. Nevertheless, the recognition on visual memory (BVMT) and the original order of Digit Span are rather simple tests which might have the results overestimated, considering that the city we held this study in has a high schooling rate. All other aspects, such as information processing speed, selective and task-switching attention, episodic and working memory, verbal fluency, and inhibition process, were all impaired on DAI group, even though it was not possible to establish a relation between the also altered SIICI values.

It seems that the attention and memory impairment in DAI are not only affected by intracortical inhibition (GABAergic) and facilitation (glutamatergic) processes but also by many other circuits with complex influences that could not be fully identified in this study. Also, CE assessments can only be performed on a single area (the motor cortex), while cognitive tasks activate different areas of the brain at the same time in a complex pattern.

Another limitation of our study for correlating CE and neuropsychological data is the limited sample size (*n* = 22), so further studies with larger sample sizes are needed to elucidate this problem. Despite there being only a few statistically significant correlation of these aspects (Table 4), post-TBI cognition recovery still needs investigation, and unfortunately, the post-trauma setting restrained knowledge on how these individuals were before the incident and on any discrepancies from the neuropsychological perspective.

When considering the control group CE data alone, some values can be interpreted as already abnormal. Still, they are not pathological and just out of what would be considered “common”/“norm.” It is worth mentioning that even in healthy individuals, CE values are subject to a large variability or be influenced by many environmental issues (lack of sleep, caffeine consumption, etc.) (17, 35). Minutely, as expected in normative data, there will be 5% of healthy sample that will not be within confidence interval (95% CI), by definition (36, 37). For this study, the control group was considered “healthy” from TBI perspective and free from any other CNS disease, so that the comparison we wanted to make was DAI patients and healthy (non-TBI) subjects.

Revising potential outliers on DAI group, they seemed mathematically outliers, however, not actual clinically outliers. This would be explained by the fact that maximum values for SIICI in healthy subjects over 50 years can be up to 6.7 and up to 3.5 for those who are under 50 years (17).

Regarding the mechanisms of TBI, Almeida-Suhett and collaborators (38) suggested that the loss of GABAergic interneurons after mild TBI reflects a reduction of neuronal inhibition. Miller and collaborators (39) also suggested an influence of mild TBI on intracortical inhibition, measured by silent period (SP) parameters. Bernabeu and collaborators (40) showed the abnormal corticospinal excitability in patients with DAI where motor recovery was related to the severity of TBI (the lower the severity, the better the motor recovery) using input–output curves and SP. These studies address mild TBI with a similar change in inhibition parameter as we found in moderate and severe TBI.

As the inhibition processes involve GABA-mediated circuitry, it is reasonable to infer that DAI pathophysiology itself (disruption of axons) may deplete GABA, contributing to a defective inhibition of neural system on the chronic phase of DAI. For better evidence of GABA depletion, we would recommend further studies measuring GABA noninvasively, if possible, using MR spectroscopy.

We acknowledge that measuring MEPs using cancelation techniques, such as the triple stimulation method, would provide better reliability and more accurate measurements of central conduction times and MEP amplitudes. However, our main aim was to have a concise and brief assessment of CE parameters that would provide a more general view of the excitability status on DAI patients. It is interesting that the present study could find altered intracortical inhibition on these patients but the imbalance of inhibition and facilitation processes is not limited only to GABA-mediated or glutamate-mediated pathways, suggesting that different mechanisms may influence on TBI recovery.

## Ethics Statement

CAPPesq—Comissao de Etica para Analise de Projetos de Pesquisa do Hospital das Clinicas da Faculdade de Medicina da Universidade de Sao Paulo. Protocol #707.642. All eligible patients were interviewed and invited to voluntarily participate in our study. All aspects of our study were explained and clarified. Those who accepted gave written informed consent upon recruitment in compliance with Declaration of Helsinki. Not applicable.

## Author Contributions

All of the authors contributed to patient recruitment, data analysis, and preparation of the manuscript.

## Conflict of Interest Statement

This research was conducted in the absence of any commercial or financial considerations that could be construed as a potential conflict of interest.
